# Kinematic characteristics of gait with different myopia: a cross-sectional study

**DOI:** 10.3389/fpubh.2023.1256242

**Published:** 2023-12-20

**Authors:** Aochuan Xue, Zhaohong Zeng, Huihui Wang, Jinming Han, Bo Pang

**Affiliations:** ^1^College of Physical Education and Health, East China Normal University, Shanghai, China; ^2^School of Sports and Health, Zunyi Medical University, Zunyi, Guizhou, China; ^3^School of Physical and Art Education, Beijing Institute of Education, Beijing, China; ^4^School of Sports Science, Harbin Normal University, Harbin, China

**Keywords:** myopia, gait, kinematics, center of gravity, joint angle, gait cycle

## Abstract

**Background:**

Myopia, a condition affecting approximately one-quarter of the world' s population, has been projected to double in prevalence by the year 2050. It can have an impact on postural control during walking and can increase the risk of falls and injuries.

**Objective:**

(1) To examine the abnormal performance of postural control during walking in male college students who used convex lenses for myopia intervention from a kinematic perspective; (2) to establish theoretical foundation for preventing falls and injuries in the myopic population.

**Methods:**

A total of 22 male college students participated in this study. The center of gravity (COG), the percentage of gait cycle (PGC) and the joint angle(JT) were collected as indications of postural control during walking. A quantitative analysis was conducted using a One-Way Repeated Measures ANOVA to examine the variations among the three groups.

**Results:**

During myopic interventions, (1) the range of vertical COG changes is significant to be greater compared with normal vision (*P* < 0.05). (2) there was an significant increase in the PGC in single-legged support, accompanied by a decrease in the PGC in double-legged support, compared with normal vision (*P* < 0.05). (3) The myopic intervention leads to increased variability in JT of the hip and the knee during the single-leg support and swing, as compared to individuals with normal vision (*P* < 0.05). Severe myopic interventions result in more changes in JT of ankle.

**Conclusion:**

Myopia has been found to have a negative impact on postural control during walking, leading to changes in balance, increased instability, and an elevated risk of injury.

## 1 Introduction

Walking is considered to be one of the most fundamental human movement abilities, requiring a vast range of coordination and control skills (1). Indeed, postural control during walking relies on the integration of sensory information from various sources, including visual, vestibular, and proprioceptive. These work together to provide the necessary feedback for maintaining balance and coordinating movements during walking. Wang et al. conducted a study on the kinematics and dynamics of the trunk and the lower limb joints during walking in three different conditions: normal posture, poor posture, and with a spinal orthosis. The results obtained indicated that poor posture, such as an abnormal joint angle, has an impact on the kinematics and dynamics of the trunk during walking, which may be due to abnormal sensory inputs altering postural control (2, 3). Vision is one of the primary sensory input organs, which effectively identifies the relative position of the body in space. When visual information is lacking, the remaining sensory information may not be adequate to maintain postural stability, resulting in reduction of postural control and lead to tumbles or injuries (4). Myopia is a visual phenomenon that causes a lack of sensory information input. Studies have shown that people with myopia are more likely to fall and suffer injuries (5). However, today, there are a significant number of studies on the changes in the physiological characteristics (6), the factors in the development of myopia (7), and the correction of visual acuity after the myopia occurs (8). Moreover, few studies have been conducted on the effects of myopia on postural control. In addition, some people with myopia only wear glasses to correct their vision at school and work, which neglects the effects of myopia on walking and sports, ultimately resulting in tumbles and injuries. It is essential to conduct this study.

The study of myopia in postural control during walking can be based on kinematic parameters. It has been demonstrated that abnormalities in body postural control, such as those seen in stroke and scoliosis patients, result in various changes in gait kinematic data (3, 8, 9). The kinematic analysis can be used to study the temporal and spatial patterns of the limb movements during walking. Injuries and tumbles may occur when there are abnormalities in kinematic indicators such as center of gravity (10), percentage of gait cycles (11), and joint movement angles (12, 13). As a result, we plan to offer a theoretical framework for kinematic analysis-based gait training for individuals with myopia. In this study, healthy male college students were chosen as research participants to exclude the influence of other sensory inputs on the experimental results. The aim of this study was to investigate the effects of different myopic states on the gait movement characteristics of male college students by using sports biomechanics, and the gait cycle was categorized into six phases. This study hypothesized that myopia would have an impact on postural control in walking, as evidenced by changes in kinematic parameters such as center of gravity, percentage of gait cycles, and joint movement angles.

## 2 Subjects and methods

### 2.1 Study design

This study can be classified as a cross-sectional randomized clinical trial.

### 2.2 Participants

30 male college students with normal vision were randomly recruited from Zunyi Medical College, and 22 of them passed the screening of the inclusion criteria to become the participants. The average age of the participants was 20.82 ± 1.40 years, the average height was 174.86 ± 3.27 cm, the average weight was 65.48 ± 9.48 kg, and the average BMI was 21.32 ± 2.54 kg/m^2^. This study has been approved by the Medical Ethics Committee of Zunyi Medical College, and the ethical number is Ethical Review of Zunyi Medical University (2022) No. 2-014. All subjects were informed about the content and procedure of the experiment and signed an informed consent form.

#### 2.2.1 Criteria for inclusion

The inclusion criteria for this study are as follows: (1) Normal visual acuity, bilateral bare eye visual acuity 5.0, (2) Normal gait without motor impairment, normal cognition capable of completing the experiment according to the instructions, (3) No recent history of refractive or ocular surgery.

#### 2.2.2 Criteria for exclusion

The criteria for this study are as follows: (1) having poor vision, with visual acuity of 5.0 in either eye, (2) experiencing motor impairment, walking impairment, or cognitive abnormalities that prevented the completion of the experiment, (3) having had recent eye surgery, such as refractive correction or other surgery that affects the vision of the eye.

### 2.3 Methods

**Figure 1:**
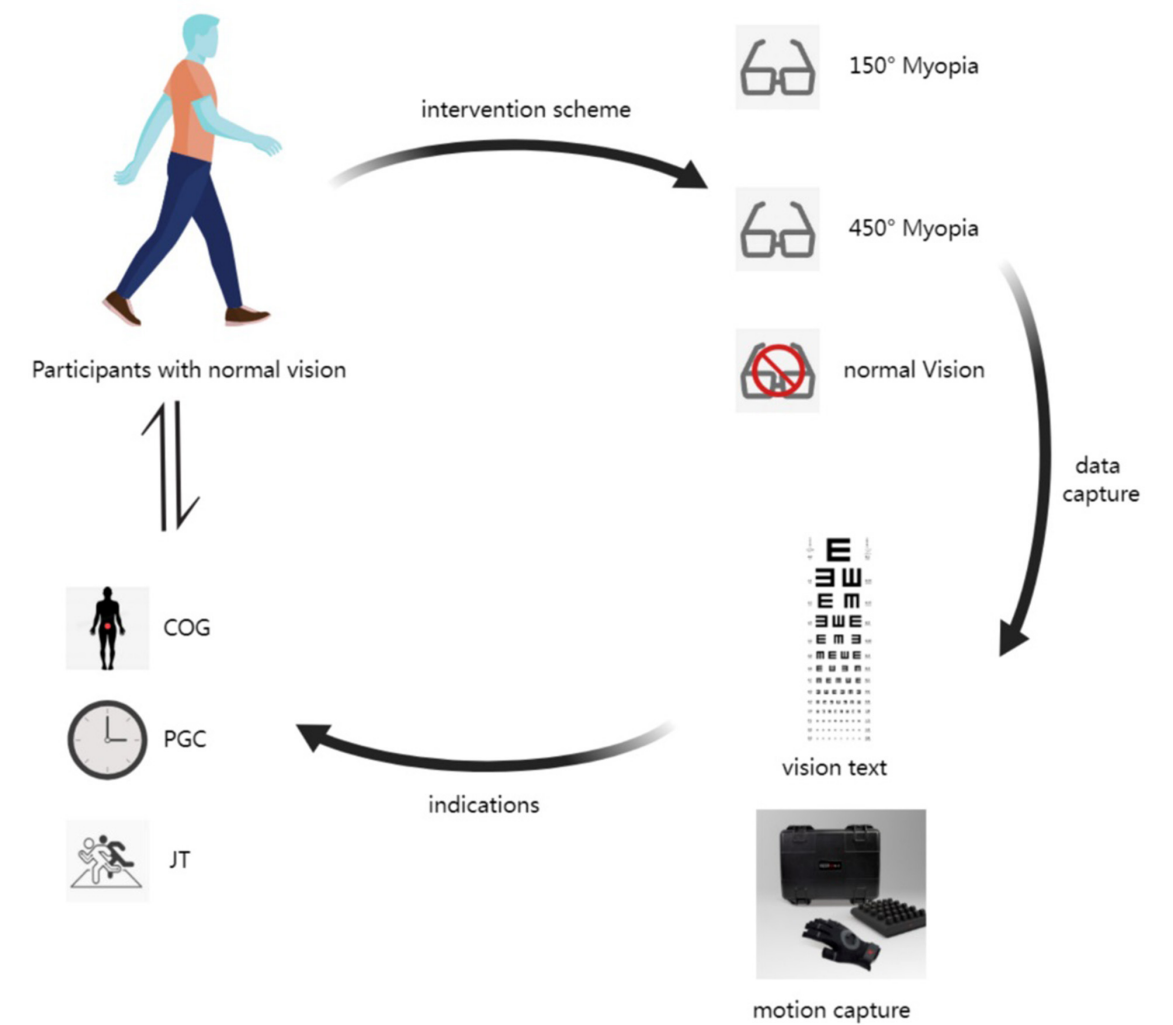
Process diagram of the experiment. The COG represents the center of gravity, the PGC represents the percentage of gait cycle, and the JT represents the joint angle.

Gait kinematic data were collected from participants with normal vision, wearing 150° and 450° convex lens interventions. One-Way Repeated Measures ANOVA was used to compare the differences in kinematic parameters among the three groups. Ultimately, this study analyzes the results of myopia affecting gait kinematic characteristics. The experimental process is shown in Figure 1.

#### 2.3.1 Data acquisition

##### 2.3.1.1 Data collection for visual acuity

The visual acuity test site must be clean, tidy, and quiet, and the size and lighting of the testing area must be appropriate to ensure accurate result. The test is conducted using a standard logarithmic visual acuity chart, which consists of 12 rows of “E” symbols of different sizes and openings in different directions. Visual acuity measurements span a numerical spectrum between 4.0 and 5.2, designated by numerals on each line. The visual acuity meter is hung at a height at which the 5.0 row sight mark is at eye level with most of the measurers. The illumination is ~300 to 500 lux (14).

Before the test, participants rested in a calm state for at least 10 min. Participants were randomly assigned to wear either 150° or 450° convex lenses to adapt to the relevant visual status. After completing the adaptation to the visual acuity state, they kept their bodies upright and were tested at a distance of 5 meters from the visual acuity charts, starting with the right eye and then the left.

Participants were instructed to cover their left eye first with an eye shield, completely shielding the left eyeball. Participants started with the largest sight mark and indicated the direction of each sight mark in turn. The participant is asked to point out the direction of the opening of the sign within 3; the visual acuity shown in the last line of the correct opening direction is the result of the visual acuity test for that eye. The procedure for the left eye is the same as for the right eye. The visual acuity was recorded using the 5-point recording method. A 5% sample of participants was selected for review after the vision test to ensure data quality (15). According to the theoretical basis, myopia is classified as mild myopia with a visual acuity above 4.9, moderate myopia with a visual acuity between 4.6 and 4.8, and severe myopia with a visual acuity below 4.5 (15). The participants wore 150° and 450° convex lenses as the intervention in this study.

##### 2.3.1.2 Data collection for gait

**Figure 2:**
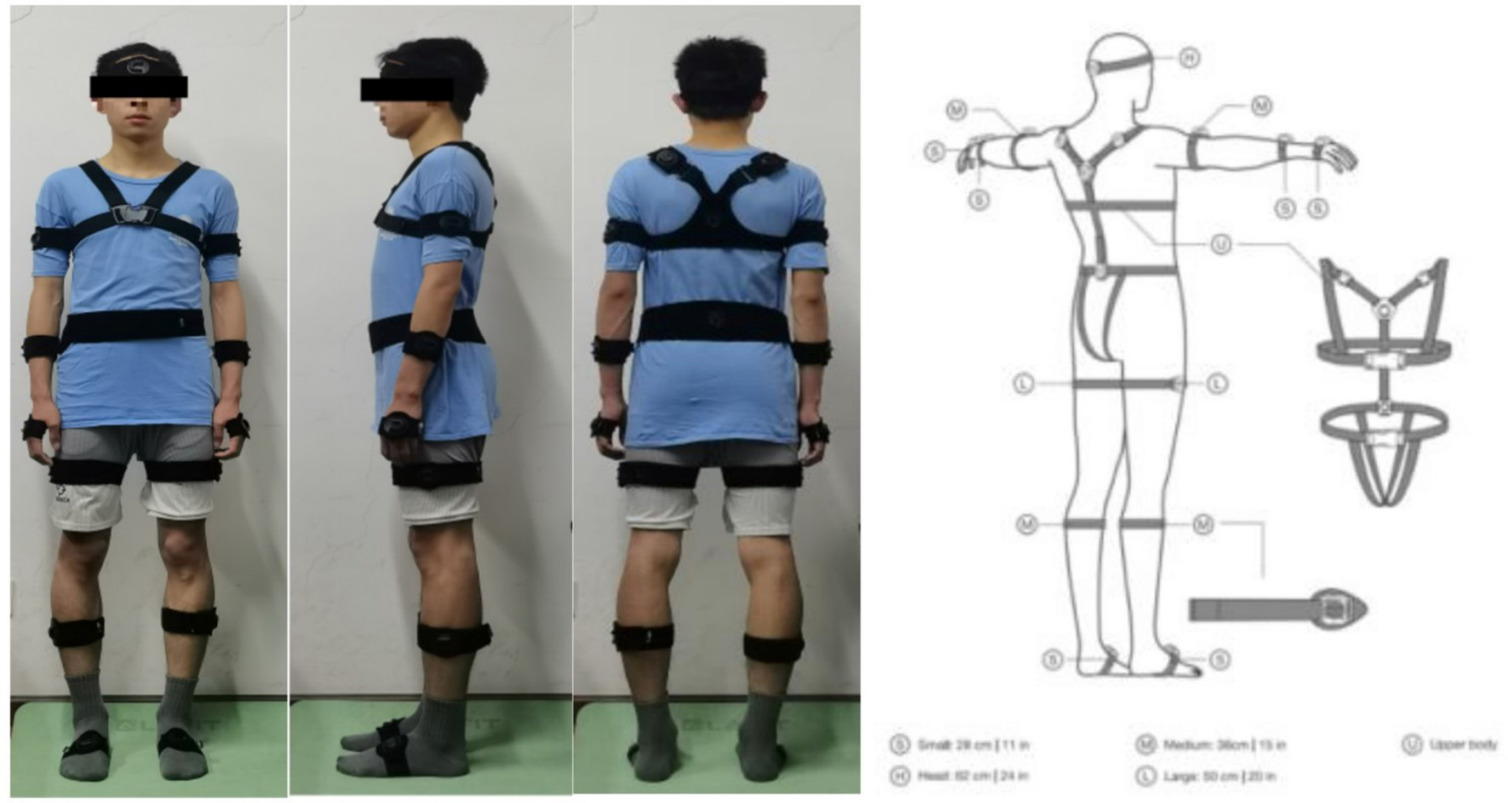
Wearing sensors.

In this study, the PN3 Pro inertial measurement unit (PN3 Pro is PERCEPTION NEURON 3, which is an inertial sensor unit for motion capture) and Axis Studio software were used to test participants' gait kinematic data. Before the test, it is important to follow these steps: 1. To activate the sensor, enter the software Axis Studio software should be accessed. First, the sensor needs to be inserted into the charging case, and then the charging case should be disconnected. This action will automatically turn on the PN3 sensor; 2. For sensor placement, a plastic chair of suitable height should be positioned at the center of the motion acquisition area, and an anti-magnetic carrying case containing the sensors and charging box stationery should be placed on the plastic chair; 3. The sensor connection should be initiated by clicking; 4. The body morphometric data of the participants were measured to generate individualized models. The morphological data included height, arm span, head height, neck length, shoulder width, trunk length, femur width, upper arm length, forearm length, hand length, thigh length, calf length, foot length, and heel height; 5. The wear locations for the sensors included the midpoint of the forehead, the upper outer third of both scapulae, the forearm, the outer midpoint of the thigh, the fourth lumbar vertebra, the outer midpoint of the thigh and calf, the back of the foot, and the back of the hand (as shown in Figure 2). A total of 16 sensors were worn, with calibration available upon request after donning (16).

After the sensors were put on and calibrated, participants walked smoothly and evenly barefoot in a defined 10^*^3 area under the guidance of an operator while randomly selecting either vision state with 150° or 450° convex lenses or without glasses. At the same time, the PN3 inertial measurement unit and Axis Studio software were used to collect gait kinematic data in three visual states. It is recommended to retake the test if the participant becomes unstable during the test. Three successes were obtained for each vision state.

The data collected by the PN3 Pro inertial measurement unit were presented in three dimensions in real time in Axis Studio software. Based on the real-time images in the software, the data of the corresponding indicators were exported according to the stages of the gait cycle. The gait cycle was staged as follow: defining a complete gait cycle as the period from the landing of the left heel to the landing of the left heel again, it was divided it into six stages. Pre-double support: left heel pointing, right toe off the ground; pre-single support: right toe off the ground, right heel passing the left foot; late-single support: right heel passing the left foot, right heel on the ground; late-double support: right heel on the ground, left toe off the ground; pre-swing: left toe off the ground, left heel passing the right heel; late swing: left heel passing the right heel, left heel pointing (11).

Collection indicators: The center of gravity (COG), the percentage of gait cycle (PGC) and the joint angle(JT) were collected as indications of postural control during walking.

#### 2.3.2 Data processing

The data were collated using Excel and analyzed using SPSS 29.0. The Shapiro-Wilk test was used to examine the normality of the data. Measures were expressed as mean ± standard deviation, and data were compared among the three groups using one-way repeated measures ANOVA with *post-hoc* comparisons using the LSD analysis (16). A repeated measures one-way ANOVA, which determines whether all the samples are the same. It is used to determine whether there are any statistically significant differences between the means of three or more independent (unrelated) groups. Level of α = 0.05 was significant.

## 3 Results

### 3.1 Visual acuity

As shown in Table 1, participants were at moderate myopia when wearing the 150° convex lens intervention and at severe myopia when wearing the 450° convex lens intervention.

**Table 1:** Visual acuity test results.

		**Visual acuity**	**Lower limit**	**Upper limit**
Vision in the left eye	Normal	5.13 ± 0.94	5.09	5.17
	150°	4.65 ± 0.19	4.57	4.74
	450°	≦4.2		
Vision in the right eye	Normal	5.15 ± 0.10	5.11	5.19
	150°	4.60 ± 0.20	4.52	4.69
	450°	≦4.2		
Vision in both eyes	Normal	5.22 ± 0.08	5.18	5.25
	150°	4.70 ± 0.18	4.63	4.79
	450°	≦4.2		

Myopic vision of 150° was tested with a 150° convex lens and myopic vision of 450° was tested with a 450° convex lens.

### 3.2 Change of center of gravity

As shown in Table 2, the change in the center of gravity in the vertical direction throughout the gait cycle was significantly greater for moderate myopia and high myopia interventions than for normal vision (*p* < 0.05).

**Table 2:** Change of center of gravity in the vertical direction.

	**Visual**		
	**Normal**	**Moderate myopia**	**Severe myopia**
Pre-DS	0.63 ± 0.20	0.60 ± 0.26	0.63 ± 0.18
Pre-SS	1.32 ± 0.30	1.34 ± 0.88	1.33 ± 0.56
Late-SS	1.76 ± 0.39	1.73 ± 0.63	1.77 ± 0.35
Late-DS	0.63 ± 0.48	0.64 ± 0.26	0.64 ± 0.18
Pre-swing	1.48 ± 0.33	1.46 ± 0.75	1.50 ± 0.29
Late-swing	1.69 ± 0.42	1.87 ± 0.35	1.93 ± 0.43
GC	2.83 ± 0.37	3.10 ± 0.48^*^	3.10 ± 0.44^#^

^*^P < 0.05; ^#^*P* < 0.05 compared with normal visual acuity.

### 3.3 Percentage of the gait cycle

As shown in Table 3, the percentage of Late-single support was significantly greater than normal vision for both moderate and severe myopia interventions (*p* < 0.05). The percentage of Late-double support is significantly smaller than normal vision (*p* < 0.05).

**Table 3:** Percentage of the gait cycle.

	**Visual**		
	**Normal**	**Moderate myopia**	**Severe myopia**
Pre-DS	8.54 ± 2.24	8.30 ± 2.44	8.14 ± 2.18
Pre-SS	18.40 ± 2.22	18.81 ± 2.49	18.73 ± 2.64
Late-SS	21.78 ± 2.10	22.98 ± 2.06^*^	23.16 ± 1.73^#^
Late-DS	8.84 ± 2.74	7.19 ± 1.86^*^	7.21 ± 2.35^#^
Pre-swing	18.78 ± 2.46	18.76 ± 1.46	18.90 ± 2.01
Late-swing	23.40 ± 1.97	23.96 ± 1.46	23.86 ± 1.95
GC	1.12 ± 0.10	1.10 ± 0.08	1.07 ± 0.07^#^

^*^P < 0.05; ^#^*P* < 0.05 compared with normal visual acuity.

### 3.4 Angle of the trunk movement

**Figure 3:**
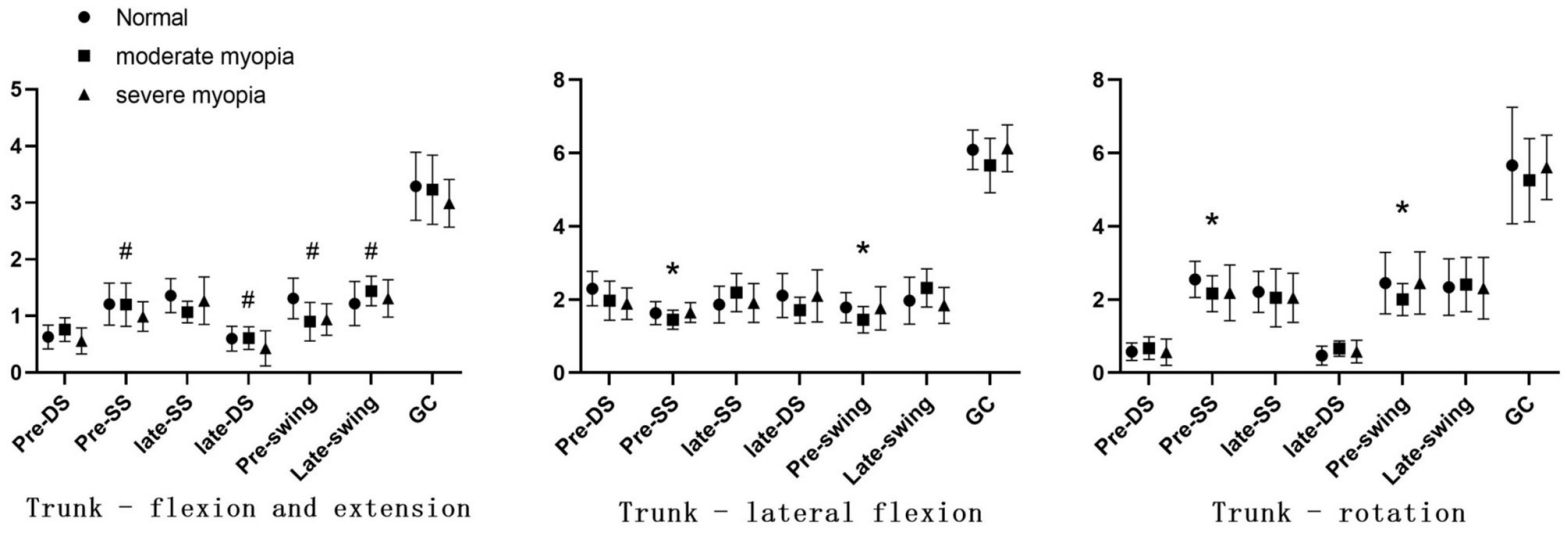
Angle of trunk movement. The DS represents the double support period, the SS represents the single support period, and the GC represents the gait cycle. *P < 0.05; ^#^*P* < 0.05 compared with normal visual acuity.

As shown in Figure 3, compared to normal vision, during the moderate myopia intervention, the angle of movement in the lateral flexion and rotation direction of the trunk significantly decreased in the Pre-swing and Pre-single support (*p* < 0.05).

During the severe myopia intervention, the angle of movement in the flexion and extension direction of the trunk significantly decreased in the Pre-single support, Late-double support, and swing phases (*p* < 0.05).

### 3.5 Joint movement angles of the hip, knee, and ankle joints

**Figure 4:**
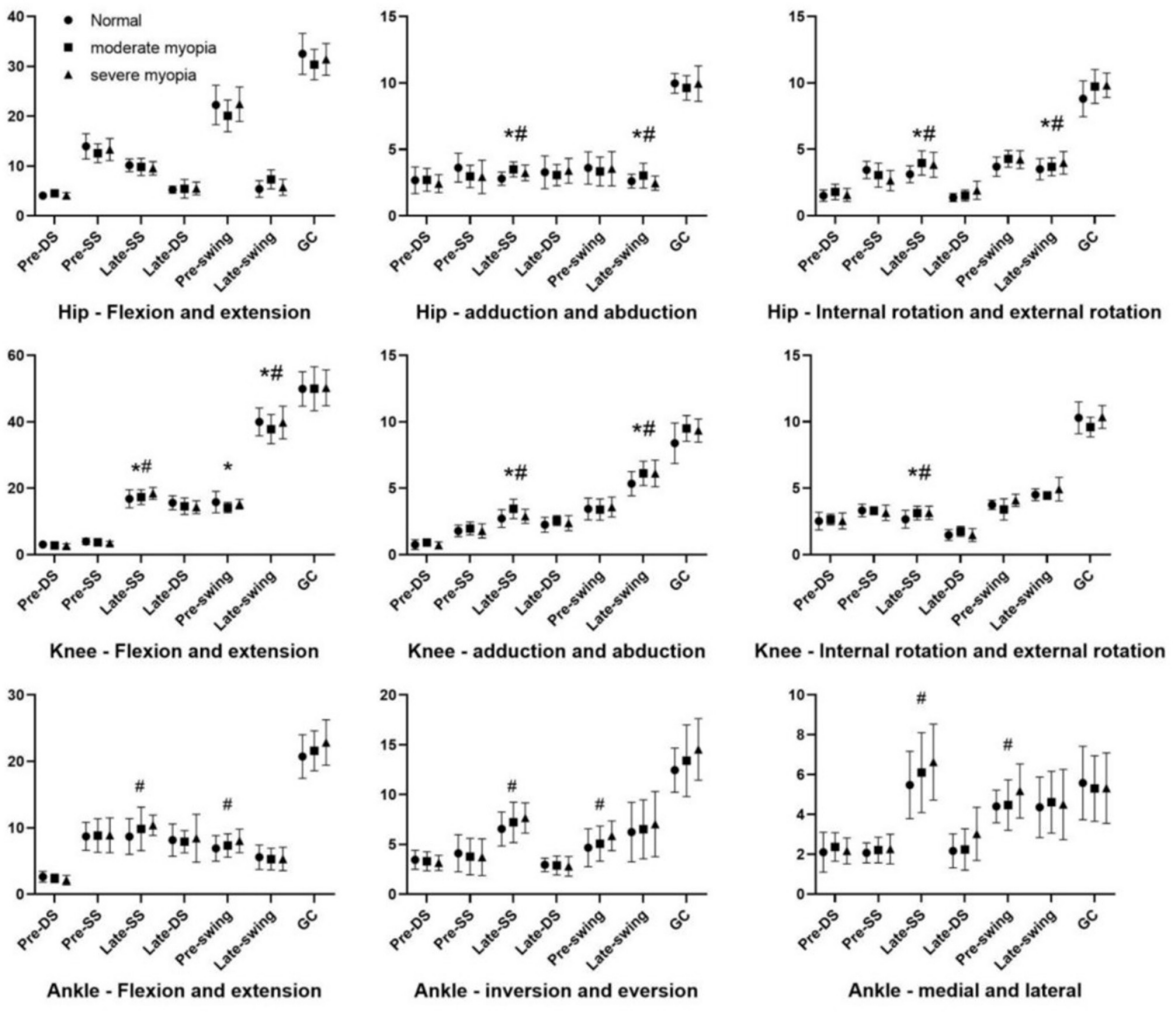
The lower limb joints' range of motion (°).The DS represents the double support period, the SS represents the single support period, and the GC represents the gait cycle. *P < 0.05; ^#^*P* < 0.05 compared with normal visual acuity.

As shown in Figure 4, compared to normal vision, during the moderate myopia and severe myopia interventions, there was a significant increase in joint movement angles as far as the direction of the hip and the knee abduction and adduction, internal and external rotation in the Late-single support (*P* < 0.05). And there also was a significant increase in joint movement angles as far as the direction of the hip abduction and adduction, internal and external rotation, and the knee abduction and adduction in the Late-swing (*P* < 0.05).

During the moderate myopia intervention, there was a significant decrease in joint movement angles in the direction of knee flexion and extension in the Late-single support and Pre-swing support (*p* < 0.05).

During the severe myopia intervention, the angle of joint motion in the direction of the knee flexion and extension significantly decreased in the Late-single support (*p* < 0.05). The ankle flexion and extension, inversion and abduction, and internal and external rotation directions of joint motion significantly increased in the Late-single support and pre-swing (*p* < 0.05).

## 4 Discussions

For the study, 22 male college students with normal visual acuity were selected. The control group for the study was individuals with normal vision. The intervention for the experimental group was to wear convex lenses of 150°and 450°. The results of the visual acuity test after the intervention are as follows: moderate myopia with 150° convex lenses and severe myopia with 450° convex lenses. There was a significant difference between the pre-intervention and post-intervention comparisons. The present experimental study affirms the hypothesized results. Myopia impairs walking postural control, resulting in an altered balance, increased instability, and increased risk of injury.

The point of gravity of an object can be defined as the center of gravity. Walking is a process in which the body's center of gravity changes, and that change in center of gravity is closely related to balance during move. A shift in the position of the body's center of gravity can disrupt the dynamic balance in gait. In addition, each float of the body's center of gravity brings about a shift between the kinetic energy of the body and the potential energy of gravity. An abnormally high change in the vertical direction of the center gravity causes an increase in energy consumption, and both the change in balance and the increase in energy consumption can thus lead to the risk of falls and injuries during gait (17). Vision affects the distribution and stability of the body's center of gravity, which in turn affects the indirect control of the trunk, resulting in an unstable balance and increased energy consumption, ultimately leading to injuries and tumbles (18). In myopic conditions, injuries and tumbles are particularly evident in participants who are involved in sports activities as time and speed in sports travel increase.

The gait cycle refers to the process of one side of the foot following the ground until the heel of that side hits the ground again. In each gait cycle, each lower limb goes through a swing phase and a support phase. The swing phase refers to the phase where the heel leaves the ground and the foot follows the ground, that is, the phase where the foot leaves the ground and takes a step forward. The support phase refers to the phase from when the foot follows the ground to when the toe leaves the ground, that is, when it is in contact with the ground and carrying weight. In the double support phase, the center of gravity is shifted from one lower limb to the other, with both lower limbs in contact with the ground at the same time. The area of support of the center of gravity is larger and more stable, which is more conducive to postural control. Although in the single-leg support phase, the area of the center of gravity's support decreases and the instability of the center of gravity increases (8). After the myopia intervention in this study, the percentage of single-leg support time in the gait of male university students increased significantly and the percentage of double-support decreased significantly. The change in the percentage of subphases of the gait cycle refers to an increase in the time of instability (13). In myopic conditions, instability increases and stability decreases in older people with degenerative walking, exacerbating the occurrence of tumbles, which, in turn, can lead to an increase in post-fall injuries. Subsequently, the possibility of fractures in older people leads to a reduced quality of life.

The trunk, as the center of the body, is the basis for supporting the movement of the limbs and the regulation of the center of gravity. Healthy gait and posture control require effective action and coordination of the lower limb and the trunk muscles (19, 20). In this study, during the moderate myopia intervention, there was a significant decrease in the trunk lateral flexion and rotation direction movement angles in the pre-single and pre-swing phases. During severe myopia interventions, there was a significant decrease in the angle of movement in the trunk flexion direction in the pre-single, late-double, and swing phases. During the moderate and severe myopia intervention stages, there was a decrease in the trunk rotation and lateral flexion angles, while the flexion and extension angles increased and decreased frequently. This indicates that people with moderate myopia may be more likely to lose their balance or fall sideways when supported on one foot and may be more likely to sprain muscles or ligaments. In contrast to moderate myopia, people with severe myopia may not be able to bend their legs enough in the late-double support phase, making it difficult for them to adapt to changes caused by terrain or other road conditions, which may also lead to tumbles or injuries during walking. In conclusion, during myopic interventions, maintaining the body stability becomes more difficult for the trunk, while controlling the posture becomes less difficult. Stability increases in the body as the trunk movement is reduced in both the vertical and coronal axes. The frequent changes in flexion and extension may be due to the body's adaptation for vertical stability of the center of gravity. This adjustment occurs through flexion and extension during myopic intervention to maintain stability and balance during walking (21).

The joint angle of motion refers to the range of motion of a joint from the beginning to the end, which reflects the flexibility and the condition of the muscles, nerves, bones, etc. (22). Gait requires the combined movement of the joints and the surrounding muscles throughout the body with more significant movements in the joints of the lower limbs. The lower limb joints consist mainly of the hip, the knee, and the ankle, and the range of motion of each joint determines the soundness of the gait (2, 23, 24).

In this study, the myopic intervention indicated an increasing trend in the direction of abduction and adduction and internal and external rotation of the hip and the knee and a decreasing trend in the direction of flexion and extension. The reduction in flexion direction refers to a change in the “soft landing” of the hip and the knee, an increase in ground reaction forces, an increase in anterior cruciate ligament loading, and an increased risk of knee injury (25). In contrast, the increase in the direction of internal abduction and internal and external rotation of each joint may be a result of the body's mobilization of hip abductor muscle groups to improve the stability in the hip, the knee, and the ankle joints. At the same time, the area of support for the center of gravity is increased and the stride length is decreased to counteract the knee instability caused by myopia (26–28). Although the body is unstable by regulating the instability by myopia, each joint has its own range of motion. Effective landings require coordinated hip, knee, and ankle movements at the same time, and changes in joint coordination strategies may also result in unstable landings, leading to injuries (29). This may be an important factor in knee injuries during sports in individuals with myopia. In this study, there were more differences in the direction of flexion and extension during the single-leg support period compared to normal gait, and the direction of adduction and abduction of the joints was also more significant during this period. The small area of support of the center of gravity during the single-leg support period was already an unstable period, and the myopic intervention exacerbated the occurrence of an injury.

Ankle joint motion angles in flexion and extension, inversion and abduction, and internal and external rotation directions exhibit different patterns during the two visual states of moderate and severe myopic intervention in the later-single support and pre-swing stages. The angles are significantly greater than those in normal vision during severe myopia but show no significant difference from normal vision in moderate myopia. It is possible that the lack of vision is more prominent during severe myopic interventions and that the body enhances the perception of movement through other sensory channels (for e.g., balance sensation and tactile sensation) to adjust the motor curves. In contrast, adaptation to lack of vision at the time of the moderate intervention may have been less, explaining why ankle angles were not significantly different from those observed in normally sighted individuals.

### 4.1 Limitations

Some of the limitations in this study are as follows: (1) Walking speed is not strictly defined in this experiment, which may influence the results obtained. (2) This experiment only selected the change of center of gravity in the vertical direction. (3) The use of concave lens for intervention in a non-myopic population. Despite providing participants with enough adaptation time, there remains a possibility that it may not be able to fully simulate the hyperopic state, potentially creating a disparity with the characteristics of a truly hyperopic population.

## 5 Conclusions

Myopia intervention leads to changes in the gait kinematic characteristics of subjects. It alters the balance in gait, decreases the stability in gait, and increases the risk of injury. Therefore, we recommend combining vision correction with gait training for people with myopia, especially those who practice sports regularly.

## Data availability statement

The datasets presented in this article are not readily available because due to our lab's policies or confidentiality agreements, we are unable to provide raw data on a temporary basis. Requests to access the datasets should be directed to Huihui Wang, 1304014776@qq.com.

## Author contributions

AX: Conceptualization, Investigation, Methodology, Project administration, Software, Visualization, Writing—original draft, Writing—review & editing. ZZ: Conceptualization, Data curation, Formal analysis, Investigation, Validation, Writing—original draft. HW: Conceptualization, Data curation, Formal analysis, Funding acquisition, Methodology, Project administration, Resources, Software, Visualization, Writing—review & editing. JH: Funding acquisition, Resources, Software, Visualization, Writing—review & editing. BP: Investigation, Methodology, Software, Validation, Data curation, Writing—review & editing.
